# Concise Review: Cell Surface *N*‐Linked Glycoproteins as Potential Stem Cell Markers and Drug Targets

**DOI:** 10.5966/sctm.2016-0109

**Published:** 2016-07-28

**Authors:** Kenneth R. Boheler, Rebekah L. Gundry

**Affiliations:** ^1^ Stem Cell and Regenerative Medicine Consortium, School of Biomedical Sciences, Li Ka Shing Faculty of Medicine, University of Hong Kong, Hong Kong, Special Administrative Region, People's Republic of China; ^2^ Department of Biochemistry, Medical College of Wisconsin, Milwaukee, Wisconsin, USA

**Keywords:** Stem cell, Pluripotent stem cells, Drug target, Technology, Proteomics, Immunophenotyping

## Abstract

Stem cells and their derivatives hold great promise to advance regenerative medicine. Critical to the progression of this field is the identification and utilization of antibody‐accessible cell‐surface proteins for immunophenotyping and cell sorting—techniques essential for assessment and isolation of defined cell populations with known functional and therapeutic properties. Beyond their utility for cell identification and selection, cell‐surface proteins are also major targets for pharmacological intervention. Although comprehensive cell‐surface protein maps are highly valuable, they have been difficult to define until recently. In this review, we discuss the application of a contemporary targeted chemoproteomic‐based technique for defining the cell‐surface proteomes of stem and progenitor cells. In applying this approach to pluripotent stem cells (PSCs), these studies have improved the biological understanding of these cells, led to the enhanced use and development of antibodies suitable for immunophenotyping and sorting, and contributed to the repurposing of existing drugs without the need for high‐throughput screening. The utility of this latter approach was first demonstrated with human PSCs (hPSCs) through the identification of small molecules that are selectively toxic to hPSCs and have the potential for eliminating confounding and tumorigenic cells in hPSC‐derived progeny destined for research and transplantation. Overall, the cutting‐edge technologies reviewed here will accelerate the development of novel cell‐surface protein targets for immunophenotyping, new reagents to improve the isolation of therapeutically qualified cells, and pharmacological studies to advance the treatment of intractable diseases amenable to cell‐replacement therapies. Stem Cells Translational Medicine *2017;6:131–138*

**Significance Statement**

Chemoproteomic techniques that target the cell surfaceome have begun to improve cell immunophenotyping, advance antibody development and usage for sorting, and identify drug targets that advance pharmacological screening and drug repurposing. The development of these techniques and application to stem cells has the potential to accelerate efforts toward the safe use of pluripotent stem cell‐derived progeny in regenerative medicine without complicating tumorigenic potential.

## Introduction

Human stem cells and their progeny are central to the advancement of regenerative medicine and cell‐based therapies of intractable syndromes like Parkinson's disease, heart failure, diabetes, and spinal‐cord injuries. The regenerative capacity of these cells relies on their ability to self‐renew and to differentiate into one or more cell types. In the clinic, their application to regenerative medicine can be credited, at least partially, to the use of immunophenotyping and cell‐sorting techniques that target mainly surface‐accessible plasma membrane proteins. These techniques permit the identification, separation, and isolation of cells according to defined properties. In the case of the prototypic hematopoietic system, only long‐term repopulating hematopoietic stem cells (HSCs), characterized by CD34^+^, CD90^+^, CD38^−^, and Lin^−^ surface proteins 1, can fully reconstitute the hematopoietic system for successful treatment of some blood disorders, cancers, and autoimmune diseases 2, 3. Similarly, mesenchymal stem cells/multipotent stromal cells (MSCs) have been extensively investigated to determine their efficacy in treating heart disease, neurodegenerative disorders, bone and cartilage defects, and other syndromes 4. Although the immunomodulatory and paracrine effects of MSCs appear promising, surface markers for immunophenotyping and sorting of MSCs are often insufficient, because processed lipoaspirate and stromal vascular fractions of adipose tissues represent highly heterogeneous cell populations that include inflammatory, endothelial , and hematopoietic cells, several of which are positive for MSC selection markers such as CD34 5. Importantly, the majority of stem or progenitor cells destined for regenerative medicine have not yet been adequately immunophenotyped for robust isolation of homogeneous cell populations suitable for clinical applications. The application of sorted stem cells and their progeny to treat many intractable syndromes, therefore, remains in its infancy.

One of the best cell sources for regenerative medicine may be embryonic stem cells (ESCs) or induced pluripotent stem cells (iPSCs). Human pluripotent stem cells (hPSCs) have the advantage in vitro of being able to differentiate into almost any cell type and can serve as an unlimited cell source for treating degenerative diseases or syndromes. Human iPSCs have overcome many of the ethical limitations associated with human ESCs (hESCs), but significant hurdles remain that preclude broad application in the clinic. Most differentiating cultures of hPSCs are heterogeneous and contain multiple cell type(s) or similar types of cells at different developmental stage(s). Moreover, these cultures may contain contaminating undifferentiated hPSCs that are tumorigenic. Cell purification techniques and elimination of tumorigenic potential usually require genetic manipulation or the use of metabolic substrates that may affect function 6. Although cell‐sorting techniques exist that are based on the presence of epitopes that enable reproducible, nonmutagenic, and high‐throughput isolation and assessments of HSC lineages 1, 3, isolation of immunophenotyped hPSC progeny that ensure both cell‐type homogeneity and stage specificity has been underutilized.

Despite their critical importance to biology, disease, pharmacological targeting, immunophenotyping, and sorting, cell‐surface proteins have been historically understudied because of technical limitations and antibody availability. Chemoproteomic techniques that target the cell surfaceome have begun to overcome these limitations by providing comprehensive analyses of surface‐accessible protein domains. Although the vast number and varied types of proteomic approaches are virtually unlimited—and undoubtedly informative—we will emphasize the utility of the cell surface capture (CSC) technology to generate experimental evidence that confirms protein identity, localization to the cell surface, transmembrane orientation, and *N*‐glycosite occupancy. Identification of *N*‐glycoproteins on stem cells and their derivatives has already begun to improve cell immunophenotyping, advance antibody development and usage for sorting, and identify drug targets that advance pharmacological screening and drug repurposing.

## Mammalian Cell Surface and Technological Advancements

Mammalian membrane proteins are structurally diverse, participate in a wide variety of biological processes, and comprise almost 22% of all proteins encoded by the genome. Among the membrane proteins identified in the genome, ∼38% have been associated with a disease 7. Some of these proteins are involved with cell‐to‐cell communication, whereas others form channels or pores to allow molecules to cross the membrane. Membrane proteins are also useful drug targets, because almost 60% of the pharmacological agents approved by the Food and Drug Administration target membrane proteins 7 8 9.

A subset of membrane proteins is located on the plasma membrane or cell surface. The vast majority (∼90%) of surface proteins have oligosaccharide (glycan) chains covalently attached either to an oxygen molecule present in an amino acid (*O*‐linked) or to an asparagine residue present on polypeptide side chains (*N*‐linked). Together with lipids, these glycoproteins form a barrier between the intracellular and extracellular compartments. Some of these proteins are exclusively localized to the cell surface, whereas others shuttle among the cell surface and intracellular compartments 10. Functionally, plasma membrane proteins participate in intercellular and intracellular communication, cellular structure, adhesion, transport, and act as environmental sensors.

Direct identification and assessment of surface proteins (i.e., the surfaceome) by using proteomic approaches have traditionally been challenging because of the relatively low abundance, hydrophobicity, and difficulty of purifying plasma membrane proteins away from intracellular membranes by biophysical methods. Innovative approaches have been used to evaluate the surfaceome based on transcriptomics 11, antibody screening, and physical and affinity enrichment strategies coupled with proteomics 12, although each has limitations. Transcript abundance does not always correlate with protein abundance on the cell surface, and transcriptomic data fail to inform subcellular localization. Although significant efforts are underway to manually curate proteomics repositories (e.g., UniProt), the precise location of many membrane proteins are as‐yet unannotated, which is further confounded by the fact that membrane proteins can be sequestered within the cell and only cycled to the cell surface in response to developmental events or cell signaling (e.g., aquaporins in kidney) 13. The orientation in which transmembrane proteins lie within the lipid bilayer is critical to the future development of new affinity reagents that recognize extracellular epitopes on live cells. For this reason, continued efforts to experimentally determine transmembrane protein orientation are needed, because predictions can be incorrect 14, 15. Although antibody‐based approaches, such as immunofluorescence imaging and flow cytometry, offer the ability to monitor protein abundance and can inform localization, they require validated and specific antibodies that are often not available for cell‐surface proteins. Moreover, determining optimal experimental conditions for each cell type of interest and validating antibody specificity requires significant time and effort. As with all antibody‐based methods, even if highly specific antibodies are available, they require the epitope to be accessible, which may not be predictable among cell types because unexpected conformational changes and posttranslational modifications can mask epitope availability.

**Figure 1:**
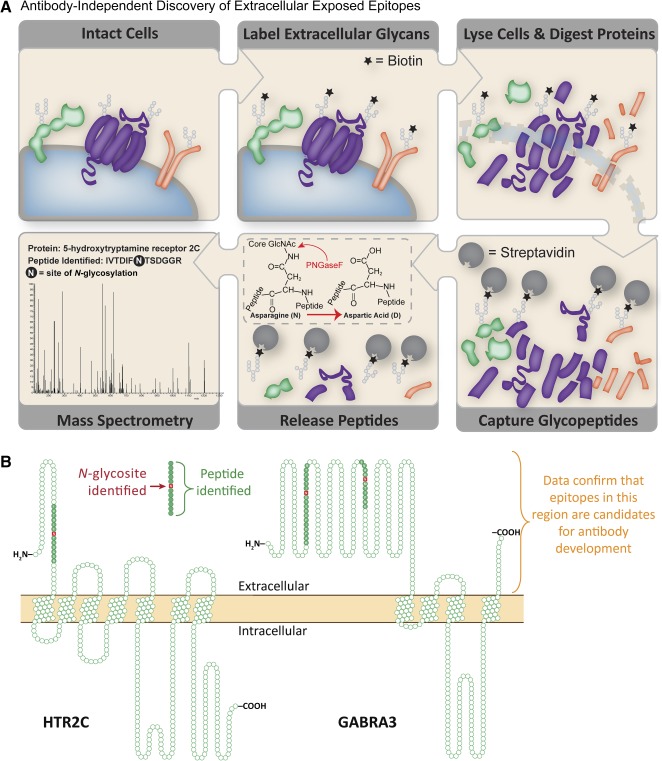
Overview of the CSC‐technology workflow and resulting data. **(A):** The experimental workflow begins with biotinylation of extracellular glycans on living cells. Subsequently, cells are lysed, proteins are enzymatically digested, and biotinylated glycopeptides are captured by using immobilized streptavidin. After extensive washing to remove nonspecific binders, *N*‐glycans were specifically cleaved from their peptide backbone via PNGaseF, which liberated the formerly *N*‐glycosylated peptides containing a deamidated asparagine. **(B):** Transmembrane protein topology is confirmed by CSC‐technology data, thereby facilitating the development of antibodies recognizing extracellular epitopes available on live cells. Transmembrane protein topology graphs are provided for 5‐hydroxytryptamine receptor 2C and γ‐aminobutyric acid receptor subunit α‐3 identified in human pluripotent stem cells via the CSC‐technology. Abbreviations: CSC‐Technology, cell surface capture technology; GABRA3, γ‐aminobutyric acid receptor subunit α‐3; HTR2C, 5‐hydroxytryptamine receptor 2C; PNGaseF, peptide‐*N*‐glycosidase F.

In contrast to the challenges faced with transcriptomic and antibody‐based approaches, the development of the chemoproteomic CSC technology has specifically enabled the discovery‐driven analysis of the cell surfaceome for a wide variety of cell types. The approach pioneered by Wollscheid et al. 16 takes advantage of the prediction that >90% of cell surface proteins are glycosylated 17, 18. Experimentally, extracellular oligosaccharides are oxidized and biotinylated by using membrane‐impermeable reagents (Fig. 1). Proteins are enzymatically digested, and the resulting biotinylated glycopeptides are enriched by using streptavidin beads. Through the actions of the enzyme peptide‐*N*‐glycosidase F (PNGaseF), which cleaves between the innermost *N*‐acetylglucosamine and asparagine residues, deglycosylated peptides are released and analyzed by high mass accuracy mass spectrometry (MS). During the data analysis process, cell surface proteins are confidently identified by the signature deamidation (+0.984 Da) observed on the asparagine residue within the conserved *N*‐glycosylation sequence motif (Asn‐*x*‐Ser/Thr/Cys [N*x*S/T/C]; where *x* is any amino acid except proline). In this strategy, the experimental output confirms the occupancy of individual *N*‐glycosylation sites of identified proteins and thereby confirms extracellular domains. Complementary variations of this approach that rely on cysteine‐ or lysine‐containing peptides have also been described 19, and quantitative assessments using label‐free and label‐based methods are beginning to be used 15, 20. Although the CSC technology method importantly offers the ability to view a highly specific “snapshot” of the cell surface at a particular time or stage—and thereby is advantageous over predictive approaches or those that rely on more generic membrane protein‐enrichment strategies—the extensive sample‐handling steps involved in the workflow ultimately result in the requirement for large amounts of starting material (e.g., 30 million to 100 million cells per experiment). Therefore, improvements that reduce the numbers of starting cells required for this approach will be critical for the future application of CSC technology to small cell populations (e.g., rare cell types or primary cells). With recent technological improvements in labeling chemistry and automated sample handling (R.L.G. and Bernd Wollscheid, unpublished data), the numbers of cells required for a CSC technology experiment are approaching 10 million to 20 million cells, and the scope is being expanded to include proteins that are exclusively *O*‐glycosylated, which are a small, but significant, population overlooked by the current method. Moreover, as with any mass‐spectrometry approach, this approach does not permit live cell recovery, and it is not yet applicable to very small numbers of cells, such as endogenous stem/progenitor cells, unless they can be expanded in vitro.

## Analysis of PSCs by CSC Technologies

Wollscheid et al. were the first to report the cell surface *N*‐glycoprotein landscape of undifferentiated and differentiating mouse ESCs (day 4, embryoid bodies; day 8, neural progenitor cells) 16. They showed that leukemia inhibitor factor receptor abundance decreased, whereas fibroblast‐like growth factor (FGF) receptor type 2 increased from days 0 to 8, thus illustrating that surface protein transitions can be informative of differentiation time. We subsequently demonstrated that cell sorting using candidate markers identified via the CSC technology could isolate iPSCs reprogrammed from mouse (m) fibroblasts. Costaining with EpCam (CD326) and PECAM1 (CD31) proved better at isolating putative iPSCs with elevated levels of Nanog, Oct4, Sal4, and Rex1 transcripts than isolation with antibodies targeting SSEA1 alone or costaining of CD112 and CD31. CD31^+^ cells sorted with CD326 were easily cultured, whereas those isolated with CD112 were difficult to maintain long‐term in culture and had low levels of Nanog transcripts. We also found that surface marker heterogeneity was greater on mouse ESCs than previously reported 21. Antibodies to EFNA2 and GPC3 had nonuniform staining in mouse ESC (mESC) colonies, whereas GP130^lo^ cells plated more efficiently and were more tumorigenic than GP130^hi^ cells. Marker selection is therefore critical for isolation of authentic iPSCs.

Although the CSC technology is highly specific, and techniques like total spectral count normalization or stable isotopic labeling by amino acids in cell culture facilitate relative quantification 20, 22, the implementation of CSC technology into a quantitative discovery workflow remains challenging and subject to strict experimental design. With this technique, only formerly *N*‐glycosylated peptides are identified. In the event that a protein has a single *N*‐glycosylation site, a single peptide will be identified. This poses challenges to obtaining robust MS‐based quantitation, which ideally relies on multiple peptides within a protein. Nevertheless, we have successfully performed quantitative comparisons between mESCs and mouse extraembryonic endoderm cells using stable isotope labeling with amino acids in cell culture (SILAC) 20. In these experiments, SILAC ratios proved consistent with flow‐cytometry analyses and, when available, were consistent with published reports. Going forward, it is likely that CSC technology approaches will continue to serve an invaluable role for the discovery of authentic surface‐accessible proteins and their initial semiquantitative assessment. Once proteins of interest are identified, applying contemporary, targeted MS‐based approaches for quantitation with sample preparation strategies that are designed to enable more complete coverage of the protein (i.e., not only the *N*‐glycopeptide) will circumvent these limitations. Targeted MS‐based quantitation strategies that accurately quantify hundreds of targets in a single experiment without antibodies 23 will be critical to the further evaluation and candidate refinement for newly discovered targets before expensive and time‐consuming antibody generation.

Ultimately, high‐quality antibodies (e.g., recombinant) that recognize extracellular epitopes on live cells are necessary to advance immunophenotyping and sorting of stem‐cell subpopulations analogous to the isolation of therapeutically relevant CD34^lo/‐^ HSC populations 1. For this reason, an added advantage of the CSC technology approach is its ability to confirm or correct predicted protein orientations 24. Protein identity, transmembrane orientation, and glycosylation site occupancy are determined simultaneously in a CSC technology experiment. As described above and as illustrated in Figure 1, high mass accuracy MS can provide unambiguous identification of peptides containing a deamidated asparagine within the NxS/T/C motif, which results from the specific capture and release of *N*‐glycopeptides during the CSC technology workflow. When this information is coupled with scanning tools that permit the selection of antigenic peptide sequences that avoid posttranslational modifications, nonspecific sequences, transmembrane regions, and signal peptides, the success in making antibodies suitable for cell sorting can be enhanced and, because only extracellular domains will be targeted, are more cost‐effective than traditional methods 25 (R.L.G., unpublished data).

## Cell Surface Protein Atlas, Immunophenotyping, and Barcodes

Extracellular epitopes from hundreds of cell surface *N*‐glycoproteins can be experimentally verified in a single CSC technology experiment. When these data are analyzed comparatively, classification of a particular protein as routinely versus rarely observed among cell types can be made immediately, thus rapidly focusing a candidate list from hundreds to tens. To enable this type of analysis, CSC technology data from more than 80 mouse and human primary cells and cell lines (normal and diseased) have now been deposited into a publicly accessible repository, the Cell Surface Protein Atlas (CSPA; http://wlab.ethz.ch/cspa) 15. The information has been highly informative to distinguish among germ layer lineages (endoderm, mesoderm, and ectoderm). Moreover, Wollscheid et al. have been at the forefront of efforts to identify markers on cancer cells that may be applicable to basic research and patient diagnoses 26 27 28 29. However, the application of the CSC technology to cancer stem cells has been limited because of the difficulty in isolating sufficient numbers of these stem cells for labeling and processing.

**Figure 2:**
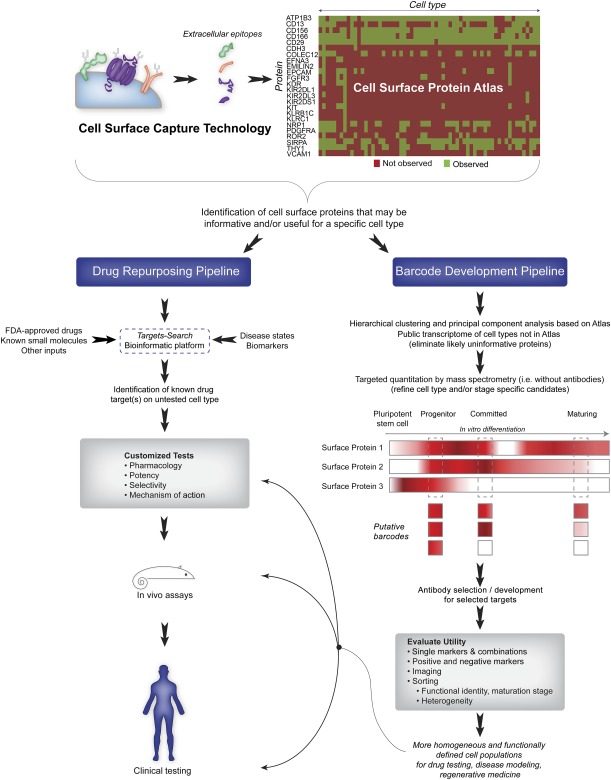
Utility of CSC‐technology to drug repurposing and barcode development. Combining the CSC‐technology with the Cell Surface Protein Atlas provides a rapid method for identifying proteins that may be informative or useful for targeting a specific cell type. Proteins identified as potentially informative can be pursued for drug repurposing or cell surface marker barcode development. In the case of drug repurposing, a new tool “Targets‐Search” should facilitate the identification of drugs for selected targets. For barcode development, hierarchical clustering can benefit the selection of marker combinations that may prove most useful. Publicly available transcriptomic data can be used to further eliminate proteins that are unlikely to be informative for a particular cell type. Subsequently, targeted mass spectrometry can quantify protein abundance of selected targets among cell types and/or stages of differentiation to further refine putative markers before antibody selection or development. Abbreviations: CSC‐Technology, cell surface capture technology; FDA, Food and Drug Administration.

With regard to blood and PSCs, the CSPA has proven instrumental in the identification of informative markers. CD29, CD90, CD156c, and CD298, for example, are observed in almost all cell types, whereas CD158 (types b2, f1, h, and i), CD159a, and CD161 are only observed in natural killer cells 15. Comparisons between human and mouse PSCs show that NRP1, COLEC12, and CD13 are restricted to mPSCs. Atlas comparisons further identified 48 markers that were unique or highly prevalent in hPSCs relative to other cell types and amenable to cell sorting (e.g., EPCAM [CD326], FGFR3 [CD333], EFNA3, and CDH3) 30. It also permitted the identification of 15 proteins that were not observed on hPSCs, but were present in human fibroblasts. Flow cytometry‐based analyses were consistent with CSC technology data, leading to the classification of these proteins as negative selection markers (e.g., CD26, CD304, and COLEC12) within the context of distinguishing hPSCs from their fibroblast precursors. As these examples illustrate, the output from CSPA‐based analyses can be utilized as a basis for generating “barcodes” that confer cell‐type identify (Fig. 2) 12. Such an approach is applicable not only to hPSCs, but to any mammalian cell type analyzed in this manner, including any type of stem/progenitor cells or their progeny. Importantly, because proteins unique to a single cell type are rare, it is expected that panels of surface markers will be necessary for adequate barcoding and sorting of well‐defined stem cell populations 15, 30.

Finally, the CSPA quantitative expression matrix permits categorization of proteins based on relative quantitative levels. This represents an efficient filtering mechanism for the identification of candidate proteins for subsequent functional studies or for evaluating surface markers identified through sorting techniques alone. CD172a (SIRPA), for example, has been described as useful for hPSC‐derived cardiomyocyte (CM) selection, but not for mPSC‐CMs 31. From CSC analyses of mouse and human cells, we observed that this protein is, in fact, present on both human and mouse PSC‐CMs. We confirmed that CD172a could be detected on mPSC‐CM by using an alternative antibody to the one used in the original study, highlighting a common challenge for antibody‐based studies, wherein the absence of a positive signal does not confirm absence of the protein, but could indicate that a conformational or posttranslational modification masks the epitope required for antibody recognition. Moreover, in our CSC technology analyses of mPSC, hPSC, and their cardiogenic derivatives (20, 21, 30 and unpublished data), we have identified previously reported cell surface markers for stem cell‐derived cardiac progenitors and cardiomyocytes—including ROR2, KDR, VCAM1 (CD106), ALCAM (CD166), ANPEP (CD13), PDGFRa (CD140a), EMILIN2, KIT (CD117), and CD172a—within a handful of experiments without the use of antibodies. Comparisons of these data within the published CSPA that have been augmented with unpublished analyses of primary hepatocytes, human iPSC (hiPSC)‐derived hepatic progenitors, pigmented retinal epithelial cells, blood cells, and cardiac fibroblasts quickly reveal that ROR2, VCAM1, ALCAM, ANPEP, PDGFRa, and SIRPA are observed across many different noncardiomyogenic cell types, which may impact their utility for identification of a single or defined cell population. Although the presence of a protein across disparate cell types may not preclude its utility for a specific context in vitro, these observations emphasize the need for extensive functional evaluations of cell populations that are identified and selected by cell surface markers.

## Drug Repurposing and Elimination of Tumorigenic Cells

Human PSCs or the presence of contaminating hPSCs in differentiated progeny is positively correlated with tumorigenesis, which represents a major hurdle to the use of hPSCs and their progeny for regenerative medicine 32. To rid differentiated hPSC progeny of any residual tumorigenic cells, researchers have used a variety of approaches, including the use of suicide genes, lactate, SSEA‐5, and Claudin‐6‐based selection approaches 6, 33 34 35 36. Although effective, each has limitations because of genetically intrusive gene delivery systems, manipulation of cells, or incomplete purity. These drawbacks have led to the suggestion that pharmacological approaches that meet Food and Drug Administration (FDA) guidelines are needed to eliminate tumorigenic potential from hPSC derivatives destined for transplantation.

The development of high‐throughput screening (HTS) platforms has been pivotal for drug discovery and for the development of novel therapies 37. The most fruitful targets of HTS platforms have been membrane proteins. Currently, almost half of the drugs that target membrane proteins are directed at rhodopsin‐like class I G‐protein‐coupled receptor superfamily members 38. The overall success of HTS platforms in identifying drug targets has in large part been because of the availability of reliable cell‐based monitoring systems for downstream outputs like calcium signals, cAMP, or cell death. The rate at which drugs are developed against new protein families, however, continues to be relatively slow because of a lack of appropriate readouts, reagent universality, cost, and confounding background signals 39.

The first success story of CSC technology data leading to drug repurposing came about through comparisons of hPSCs to other cell types within the CSPA 30. In that study, we focused on solute carrier proteins, including members (GLUT1, 3, and 4) of the glucose transporter superfamily. Although it is not possible to make a direct correlation of protein abundance among different proteins, the observation that GLUT1 was detected by significantly more spectra (hundreds) than GLUT3 and GLUT4 (tens) suggested that GLUT1 may be more abundant and the critical transporter for glucose in hPSC. Because a number of inhibitors had been identified that were reportedly “selective” for GLUT1 in cancer 40, this target protein was chosen for proof‐of‐principle experiments.

Two reported small‐molecule inhibitors of GLUT1, including STF‐31 and WZB117, and glucose deprivation were tested for toxicity in hPSCs in direct comparison with PlurSIn1, which previously had been identified through HTS techniques by Ben‐David et al. 41, 42. In these experiments, glucose deprivation proved highly toxic to subconfluent hPSCs, but toxicity was delayed and decreased in confluent cultures of hPSCs 30, 42. WZB117, an irreversible GLUT1 inhibitor, and PluriSIn1 were highly toxic to hPSCs at subconfluency, but at high densities, these small molecules did not induce significant cell death. WZB117 and PluriSIn1 were more rapid than STF‐31 at inducing hPSC toxicity in low‐density cultures, whereas only STF‐31 was effectively toxic to high‐density hPSC cultures. STF‐31 treated cocultures of hPSC and human fibroblasts or hPSC and hPSC‐CM (seeded at varying density) selectively killed hPSCs, and in teratoma assays (unpublished data), STF‐31 (10 μM) treatments of >24 hours are required to prevent tumor formation in nonobese diabetic‐severe combined immunodeficient mice, irrespective of plating density, whereas shorter treatments (e.g., 18 hours) do not prevent the formation of teratomas. Although inhibition of oleic acid biosynthesis, glucose deprivation, and GLUT1 inhibition are toxic to hPSCs in low‐density cultures, only treatment with STF‐31 showed toxicity at high cell density.

Although STF‐31 was originally reported to be an inhibitor of GLUT1, we determined that STF‐31 mediates toxicity through inhibition of nicotinamide phosphoribosyltransferase, an enzyme required for one of the NAD salvage pathways and biologically critical for normal hPSC function (see 42 for details). These results suggest that targeted proteomic analyses of cells grown under a variety of culture conditions can be an important complement to HTS studies, especially considering the technical challenges related to controlling cell density and cellular responses to stress in high‐throughput culture formats (e.g., 96‐ and 384‐well plates). Altogether, these studies indicate that metabolic strategies can be successfully used to prevent teratoma formation without genetic manipulation, cell sorting, and before cell transplantation.

## Identification of Cell Surface Targets for Enhanced Therapeutic Applications

Application of CSC technology for specific identification of cell surface proteins combined with CSPA comparisons 30, 42 enables the selection of protein targets for drug repurposing without costly HTS screening. To facilitate these types of analyses, we have developed and are beginning to test a new web‐based platform called Targets‐Search that directly links known drugs and small molecules with surface proteins identified either experimentally by CSC technology or predicted from transcriptomic data (K.R.B. and Bin Yan, manuscript in preparation). This web‐based server collects published and otherwise publicly available bioinformatics data for genes, proteins, and drugs, and is capable of associating each of these with human diseases. This platform is designed to serve as a comprehensive resource center for integrated and systemic exploitation of multimolecule interactions among drug targets, protein networks, human diseases, and cancer. As an exemplar, we have reanalyzed the hPSC cell surface proteome and were able to identify 101 proteins that are targets of FDA‐approved drugs. We also identified another 55 proteins that may be targeted by some of the 12,257 small molecules available at the Therapeutic Targets Database ( http://bidd.nus.edu.sg/group/cjttd) and the European Molecular Biology Laboratory ChEMBL database ( http://www.ebi.ac.uk/chembldb). Included among the identified targets are G‐proteins, tyrosine protein kinases, solute carrier family proteins, and transmembrane transporters. Several of the identified drugs/small molecules specifically target tyrosine protein kinases and ABCC4 transporters 43 and are being tested to determine whether they modulate signaling processes in hPSCs and their derivatives, and whether these kinases/transporters represent viable therapeutic targets. If these drugs lead to altered metabolism and differentiation, we expect that this integrated resource will be complementary to existing HTS technologies and that the output will facilitate drug repurposing efforts and enhance our biological understanding of stem cells and their differentiation in vitro (Fig. 2).

## Conclusion

Contemporary mass spectrometry‐based proteomic methods are poised to make a rapid and significant impact on the use of stem cells and their derivatives for regenerative medicine. In this review, we have emphasized the utility of the CSC technology for the generation of experimental evidence that confirms protein identity, localization to the cell surface, transmembrane orientation, and *N*‐glycosite occupancy, and how these results can foster antibody development and drug repurposing. Although we have focused on an analysis of human and mouse pluripotent cells, the strategies described here are universally applicable to any stem cell, progenitor cell, or their derivatives. Analogous to the development of immunophenotyping and sorting techniques for HSCs, the merging of broad‐based stem cell analyses with the CSC technology, CSPA, and a new bioinformatics platform for drug target screening is likely to advance our understanding of stem cell biology and accelerate applications of stem cells to basic research. The path toward cell‐based therapies and the treatment of intractable diseases will be significantly accelerated, once the research community and clinicians possess sufficient numbers of well‐defined and plentiful immunophenotyping tools for stem cells and their progeny.

## Author Contributions

K.R.B. and R.L.G.: conception and design, financial support, manuscript writing, final approval of manuscript.

## Disclosure of Potential Conflicts of Interest

K.R.B. is project coordinator for the Hong Kong Government (Innovation and Technology Commission)‐NovoHeart funded project with no overlap to this article and is an uncompensated consultant for Entopsis Asia. (This advisory/consultancy role is for cancer diagnostics, and there is no overlap with this article.) The other author indicated no potential conflicts of interest.
